# Supplementary material for: Thioflavin T indicates mitochondrial membrane potential in mammalian cells
Source: Biophys Rep (N Y). 2023 Oct 31;3(4):100134. doi: 10.1016/j.bpr.2023.100134 (PMC10679866; doi:10.1016/j.bpr.2023.100134)
Supplement: Document S1. Figures S1–S9 [file mmc1.pdf]

**Biophysical Reports, Volume *3***

## Supplemental information

# Thioflavin T indicates mitochondrial membrane potential in mammalian cells

**Emily Skates, Hadrien Delattre, Zoe Schofield, Munehiro Asally, and Orkun S. Soyer**

**SUPPORTING MATERIAL**

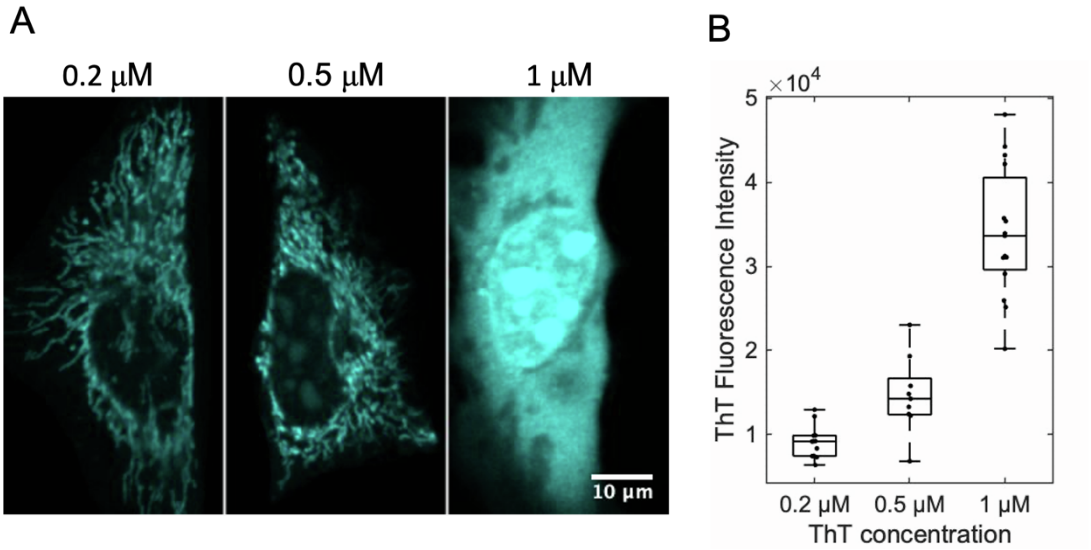


**Figure S1: (A)** Distribution of ThT in HeLa cells at ThT concentrations of 0.2, 0.5, and 1 µM (left to right). The scale bar applies to all images. Excitation was at 405 nm (see *Methods* for further details of imaging conditions). Each cell is representative of the total population. **(B)** Fluorescence intensity of a population of cells at ThT concentrations of 0.2, 0.5 and 1 µM. A total of 12, 9 and 15 cells were analysed at the respective concentrations. Differences among populations were statistically significant (U test, $p < 0.05$).

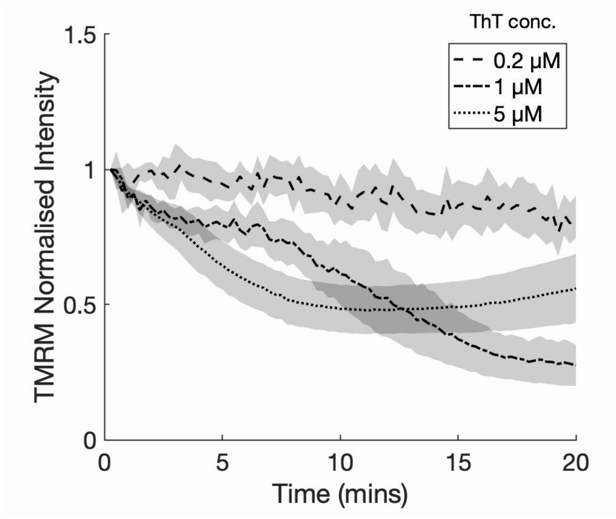


**Figure S2:** Shows the raw data from Fig 3B without background subtraction.

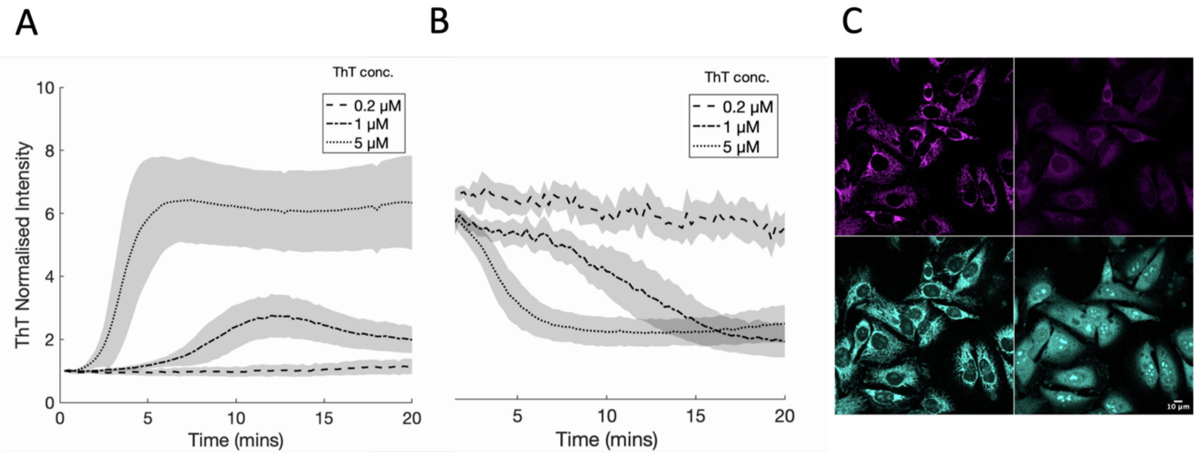


**S3:** Nuclear ThT fluorescence **(A)** and cellular TMRM fluorescence **(B)** for cells co-stained with 25 nM TMRM and 0.2, 1 and 5 µM ThT and imaged in red and blue light**.** On each panel, the bold lines show the mean fluorescence intensity, and the shaded section shows population standard deviation, SD (mean$\pm$SD). Data is collated from three independent experiments, each with three technical repeats resulting in a total of 177 cells analysed. **(C)** Snap shot images at the beginning and end of the experiment shown on panels A and B, with 5 µM ThT. The top two images is the TMRM fluorescence taken at the beginning (left) and end (right) of the timelapse. The two bottom images is the ThT fluorescence taken at the beginning (left) and end (right) of the timelapse.

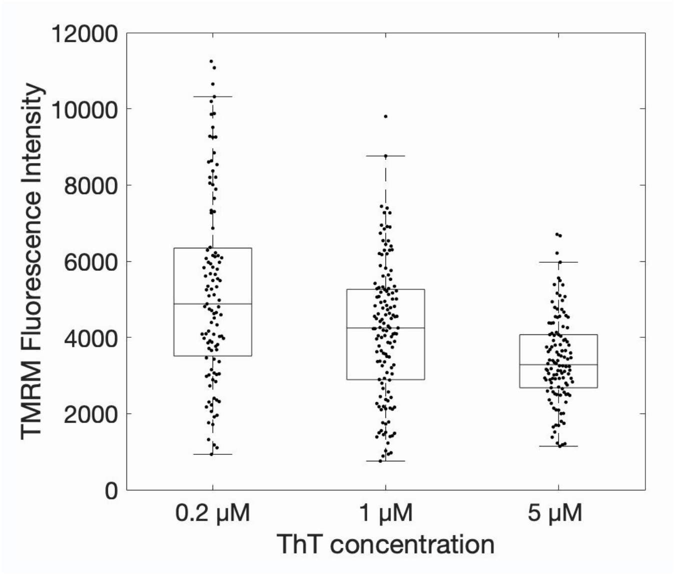


**Figure S4:** The response of cellular TMRM fluorescence (25 nM) after co-staining with 0.2, 1 and 5 µM ThT for 1 hr. Each data point represents one cell, with the analysed population for the three ThT concentrations totalling 107, 133, and 118 cells respectively.

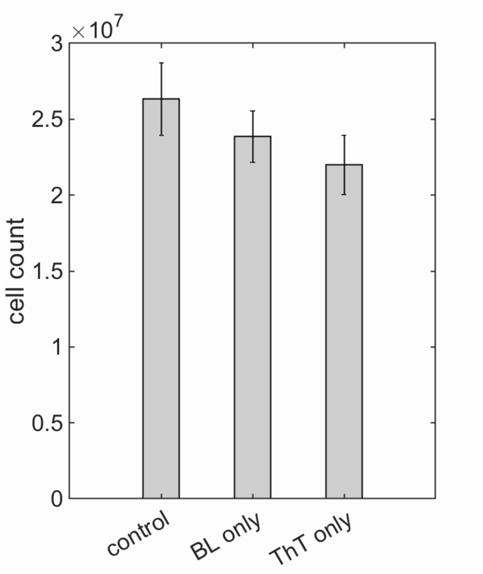


**Figure S5:** Cell count data 24hrs after HeLa cells were incubated on their own (control), with 5 μM ThT in the dark (ThT only), or exposed to 5 minutes of blue light (~84.7uW, BL only). There were no significant differences observed among these three experimental groups ($p < 0.05$). Data was collated from three independent experiments, each with three technical repeats.

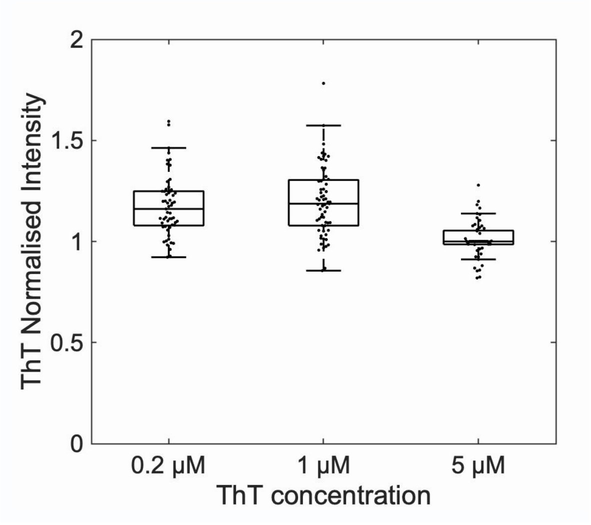


**Figure S6:** Cellular ThT fluorescence when cells were co-stained with TMRM (25 nM) and ThT (0.2, 1 and 5 μM). ThT fluorescence intensity ratio between before and after cells were imaged in red light for the time-lapse experiment shown in Fig 3C, i.e. beginning and end points of the experiment.

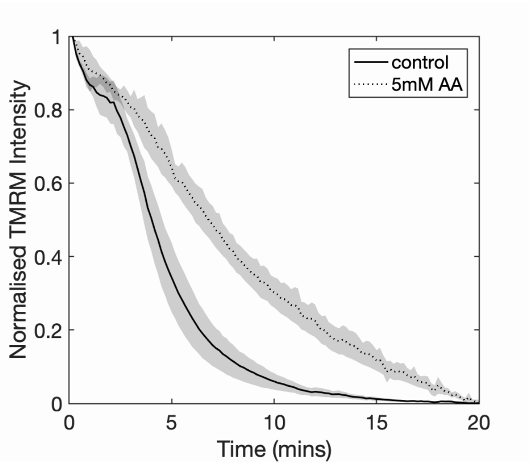


**Figure S7:** The effect of ascorbic acid on ThT-blue light induced TMRM signal loss. The average fluorescent trace of HeLa cells incubated with 5 µM ThT, 25 nM TMRM, and with and without 5 mM of ascorbic acid (AA). The black lines represent the mean fluorescence intensity with the shaded section showing population standard deviation, SD (mean ± SD). Data is collected from three independent experiments, each with three technical repeats resulting in a total of 137 cells for the control and 160 cells in the case of ascorbic acid treatment.

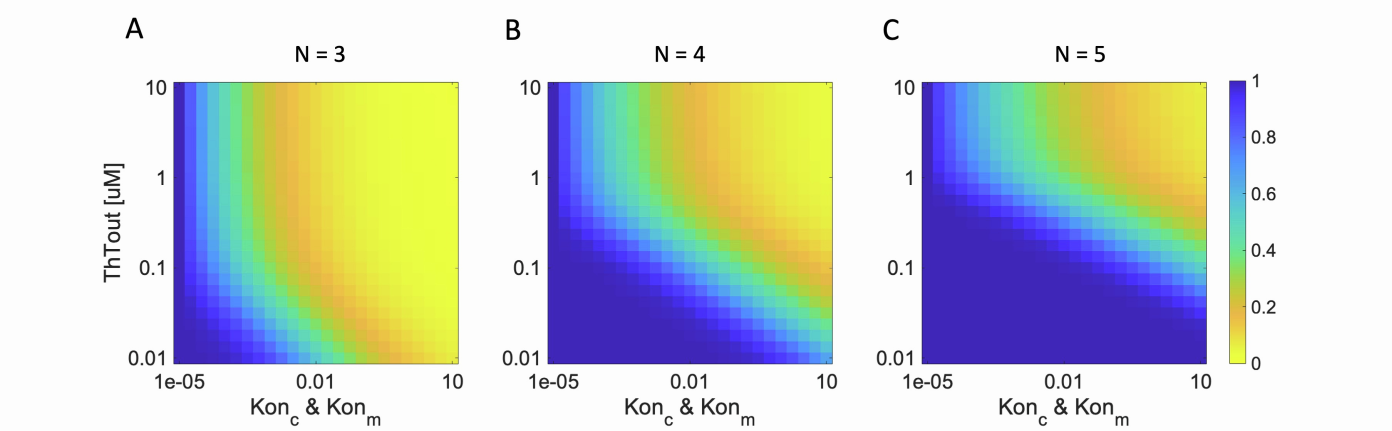


**Figure S8:** Phase plots showing normalised steady-state TMRM level (colour coded) from simulations performed with different levels of ThT photosensitisation (x-axis, parameters $Kon_c$ and $Kon_m$, mimicking increasing photosensitisation in the cytosol and mitochondria respectively) and different amounts of ThT (y-axis, ThTout). **A-C)** Phase plot results for different values of the model parameters controlling the coefficient of nonlinearity in the Hill-function used for modelling the photosensitisation effect (*N*).

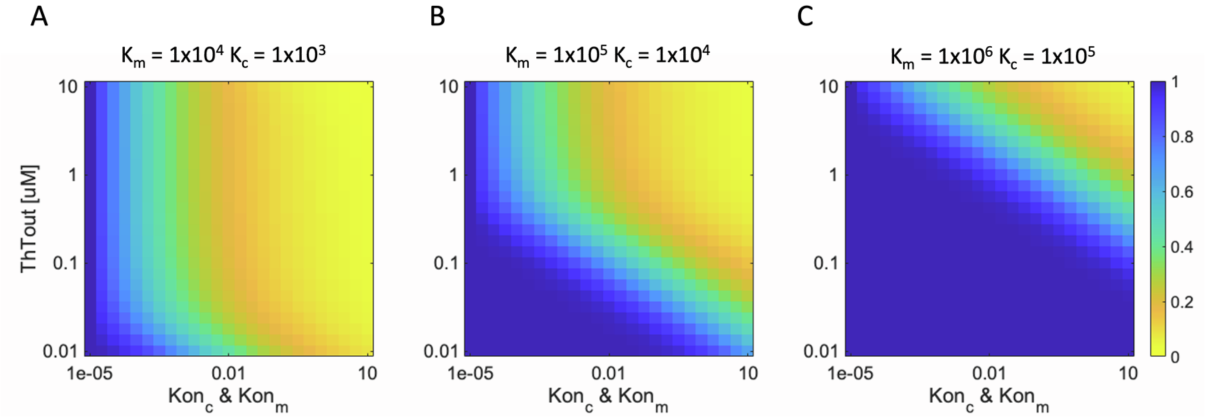


**Figure S9:** Phase plots showing normalised steady-state TMRM level (colour coded) from simulations performed with different levels of ThT photosensitisation (x-axis, parameters $Kon_c$ and $Kon_m$, mimicking increasing photosensitisation in the cytosol and mitochondria respectively) and different amounts of ThT (y-axis, $ThT_{out}$). **A-C)** Phase plot results for different values of the model parameters controlling the saturation concentration for the Hill-function used for modelling the photosensitisation effect ($K_m$ and $K_c$, for photosensitisation dynamics in mitochondria and cytosol respectively).
