# Supplementary material for: Thioflavin T indicates mitochondrial membrane potential in mammalian cells
Source: Biophys Rep (N Y). 2023 Oct 31;3(4):100134. doi: 10.1016/j.bpr.2023.100134 (PMC10679866; doi:10.1016/j.bpr.2023.100134)
Supplement: Document S2. Article plus supporting material [file mmc2.pdf]

Biophysical Reports

ARTICLE

Thioflavin T indicates mitochondrial membrane potential in mammalian cells

Emily Skates,[1,2,3,4] Hadrien Delattre,[2] Zoe Schofield,[1,2,3] Munehiro Asally,[1,2,3,*] and Orkun S. Soyer[1,2,3,*]
[1]Bio-Electrical Engineering Innovation Hub, University of Warwick, Coventry, United Kingdom; [2]School of Life Sciences, University of Warwick, Coventry, United Kingdom; [3]Warwick Integrative Synthetic Biology Centre (WISB), University of Warwick, Coventry, United Kingdom; and [4]Midlands Integrative Doctoral Training Program; University of Warwick, Coventry, United Kingdom

ABSTRACT The fluorescent benzothiazole dye thioflavin T (ThT) is widely used as a marker for protein aggregates, most commonly in the context of neurodegenerative disease research and diagnosis. Recently, this same dye was shown to indicate membrane potential in bacteria due to its cationic nature. This finding prompted a question whether ThT fluorescence is linked to the membrane potential in mammalian cells, which would be important for appropriate utilization of ThT in research and diagnosis. Here, we show that ThT localizes into the mitochondria of HeLa cells in a membrane-potential-dependent manner. Specifically, ThT colocalized in cells with the mitochondrial membrane potential indicator tetramethylrhodamine methyl ester (TMRM) and gave similar temporal responses as TMRM to treatment with a protonophore, carbonyl cyanide-4-(trifluoromethoxy) phenylhydrazone (FCCP). Additionally, we found that presence of ThT together with exposure to blue light ($\lambda = 405$ nm), but neither factor alone, caused depolarization of mitochondrial membrane potential. This additive effect of the concentration and blue light was recapitulated by a mathematical model implementing the potential-dependent distribution of ThT and its effect on mitochondrial membrane potential through photosensitization. These results show that ThT can act as a mitochondrial membrane potential indicator in mammalian cells, when used at low concentrations and with low blue light exposure. However, it causes dissipation of the mitochondrial membrane potential depending additively on its concentrations and blue light exposure. This conclusion motivates a re-evaluation of ThT's use at micromolar range in live-cell analyses and indicates that this dye can enable future studies on the potential connections between mitochondrial membrane potential dynamics and protein aggregation.

WHY IT MATTERS In mammalian research, thioflavin T (ThT), a cationic dye, serves as a prominent marker for protein aggregates; however in bacterial cells, it has been used as a membrane potential indicator. We show here that ThT acts as a mitochondrial membrane potential indicator in mammalian cells, when used at low concentrations and with low blue light exposure. However, it causes dissipation of the mitochondrial membrane potential depending additively on its concentration and blue light exposure. This conclusion motivates a re-evaluation of ThT's use at micromolar range in live-cell analyses and indicates that this dye can enable future studies on the potential connections between mitochondrial membrane potential dynamics and protein aggregation.

INTRODUCTION

Thioflavin T (ThT) is a fluorescent cationic benzothiazole dye that is widely used for quantification of amyloid fibril aggregation (1). These aggregates are shown to associate with a wide range of diseases such as Alzheimer's, Parkinson's, type II diabetes, and other age-related degenerative diseases (2,3), resulting in the wide use of ThT as a marker and research tool for studying these diseases (4,5,6). The structure of ThT consists of a dimethylated benzothiazole ring coupled to a dimethylamino benzyl ring. In solution, these two rings act as a molecular rotor, and their rotation around each other causes the low fluorescence emission of free ThT (7,8). Amyloid fibrils offer ThT a binding site, immobilizing its rotation, and thereby causing a characteristic increase in its fluorescence (9). ThT, by the same mechanism, can also exhibit

Submitted August 14, 2023, and accepted for publication October 25, 2023.

*Correspondence: m.asally@warwick.ac.uk or o.soyer@warwick.ac.uk

Editor: Jorg Enderlein.

https://doi.org/10.1016/j.bpr.2023.100134

© 2023 The Authors.

This is an open access article under the CC BY license (http://creativecommons.org/licenses/by/4.0/).

increased fluorescence by binding to DNA (10) and RNA (11) and by forming micelles (12).

Within the bacterial research community, it was shown that ThT can act as a membrane potential indicator (13, 14) and can also influence bacterial membrane potential under certain conditions (15, 16). In *Bacillus subtilis*, ThT distribution across the cell mimics that of 3-3′-dipropylthiadicarbocyanine iodide ($DiSC_3(5)$), an established reporter for bacterial membrane potential (13,17), and of tetramethyl rhodamine, methyl ester (TMRM), a mammalian mitochondrial membrane potential dye that has also been used with bacteria (16,18). These findings prompt a question whether the intracellular ThT distribution in mammalian cells may also follow mitochondrial membrane potential ($\Delta\Psi m$), which would be important for appropriate utilization of ThT in mammalian cell research and diagnosis. Previous studies have shown that ThT can localize in mitochondria and nucleoli of mammalian cells (19,20,21), but these studies did not consider any $\Delta\Psi m$ dependence of ThT distribution.

Historically, the Nernstian equilibrium distribution of cationic lipophilic dyes, such as tetra-phenylphosphonium (22) and the rhodamine dyes; e.g., rhodamine 123, TMRE, and TMRM (23,24), have been used as markers for $\Delta\Psi m$ in mammalian cells. Nernstian sensors are positively charged molecules that can diffuse across biological membranes. Therefore, they accumulate in the mitochondrial matrix according to the electric potential across the mitochondrial inner membrane. By using the Nernst equation, which relates the electrical potential gradient (membrane potential) to the concentration gradient, the fluorescence intensity of the dye can be related to the $\Delta\Psi m$. Therefore, higher $\Delta\Psi m$ leads to stronger fluorescence signal from mitochondria, whereas a decrease in $\Delta\Psi m$ results in reduced fluorescence. Any factors or conditions that alter $\Delta\Psi m$, such as changes in metabolic activity or production of reactive oxygen species (ROS), can result in changes in the fluorescence intensity of dye. It must also be noted that Nernstian dyes, being charged molecules themselves, can directly impact membrane potential under some instances.

Although plasma and mitochondrial potential are believed to be the main driver of the cellular distribution of Nernstian dyes, both dye distribution and fluorescence can also be altered upon binding to cellular components and through direct or indirect effects of this on $\Delta\Psi m$ (23,25). For example, binding of the dyes to mitochondrial components can reduce the respiration activity, which suggest possible disruption of $\Delta\Psi m$ by the dyes (24). Among the cationic dyes for $\Delta\Psi m$, TMRM is most widely used due to its low nonspecific binding to cellular components and its low effects on cell physiology, its high fluorescence signal, and its rapid and reversible equilibration across the membranes (24). Given its cationic nature, ThT would also be expected to distribute itself in cells according to the Nernst equation, but possibly with its distribution also influenced by its ability to bind to macromolecules such as protein aggregates, DNA, and RNA. It is also possible that such nonspecific binding of ThT would impact $\Delta\Psi m$ through direct and indirect effects.

Here, we analyzed ThT dynamics in HeLa cells as a model mammalian system. We found that ThT, when applied at low micromolar concentrations and with low blue light ($\lambda = 405$ nm) exposure, distributes in the cell according to $\Delta\Psi m$. In particular, ThT co-localized in cells with TMRM and gave similar temporal responses as TMRM to the perturbance of $\Delta\Psi m$. With increased concentrations, and when cells were under high blue light exposure, ThT also caused a depolarization of the mitochondrial membrane. These observations were recapitulated by a simple mathematical model that incorporates potential-dependent distribution of ThT and assumes a light- and ThT-dependent mitochondrial depolarization. Taken together, these results show that ThT can act as a $\Delta\Psi m$ indicator dye in mammalian cells but can also dissipate $\Delta\Psi m$ in a manner dependent on both concentration and blue light. Although the latter finding cautions against the use of ThT for live cell imaging at high concentrations and under high light exposures, the former finding opens the possibility for utilizing ThT for studying $\Delta\Psi m$ dynamics.

MATERIALS AND METHODS

Cell culture

The HeLa cells were sourced from the Public Health England (ECACC catalog no. 93021013). The cells were kept as cryo-stocks and live cultures, where the latter was never passaged more than 10 times or for longer than 3 months as stated in the guidelines for cell culturing (43). Cultures were maintained in minimum essential media with $NaHCO_3$ (Sigma Aldrich, M2276), supplemented with 1% L-glutamine (Sigma Aldrich, G7513), 10% heat-inactivated fetal calf serum, 1% nonessential amino acids (Sigma Aldrich, M7145), and 1% penicillin/streptomycin solution (Sigma Aldrich, P4333) and stored in a humidified atmosphere in 5% CO_2 at 37°C. Cells for fluorescence microscopy were seeded at 2×10^5 in glass bottom six-well plates (MatTek, P06G-1.5-10 F) and cultured until ~70% confluency. For microscopy, cells were first washed with PBS and then placed in minimum essential media without $NaHCO_3$ and buffered with 10% HEPES (Sigma Aldrich, H0887) instead and supplemented with 10% heat-inactivated fetal calf serum (Thermo Fisher Scientific, 1008-2147) and 1% penicillin/streptomycin solution (Sigma Aldrich, P4333).

For cell viability experiments, cells were seeded at 2×10^5 in glass bottom six-well plates and cultured until ~70% confluency. Cells

were then exposed to either blue light (for 5-min duration at ~84.7 μW and 405 nm) or incubated with 5 μ M ThT and then incubated for 24 hrs. A cell count was then performed using a heamocytometer where dead cells were excluded using trypan blue (Thermo Fisher Scientific, 15250061).

Dyes and chemical reagents

Thioflavin T (ThT, Sigma Aldrich T-3516) was kept as a 10 mM stock solution in distilled water and stored at 4°C in the dark. TMRM (Thermo Fisher Scientific, T-668) was dissolved in DMSO to make a 10 mM stock, which was stored at –20°C in the dark. Carbonyl cyanide 4-(trifluoromethoxy)phenylhydrazone (FCCP, Abcam ab120081) was dissolved in DMSO to make a 1 mM stock and stored at –20°C in the dark. Ascorbic acid (Sigma Aldrich, A4403) was dissolved in distilled water to make a 1 M stock solution, which was made fresh before every experiment.

Fluorescence microscopy

All images and time-lapse videos were recorded using a laser scanning confocal microscope (LSM-880, Zeiss, Oberkochen, Germany) with an EC Plan-Neofluar 40x/1.30 Oil DIC M27 objective lens unless stated otherwise. After the addition of either fluorescence dye, cells were incubated in HEPES buffered media at 37°C with ambient atmosphere for an hour in the dark to allow for the equilibration of the dye and cells. ThT and TMRM dyes were imaged using a 405- and 561-nm laser, respectively. The maximum power output for both lasers was 250 mW, and they were applied at a 5% power setting. Light intensity was measured at the sample plane using a power meter (PM160T, Thorlabs, New Jersey) for each laser, giving 48.4 and 27.7 μ W for the 561- and 405-nm laser, respectively.

For the assessment of the localization of ThT dye at different concentrations, cells were incubated with 0.2, 1, and 5 μ M of ThT for an hour before imaging. For the co-localization assay, cells were incubated for an hour with 25 nM TMRM and 0.2 μM ThT before imaging. For these experiments, cells were imaged using a Plan-Apochromat 63x/1.40 Oil DIC M27 objective lens (Zeiss, Oberkochen, Germany), and frame averaging was set to 16 to improve signal/noise ratio (i.e., 16 images were taken and averaged over each pixel).

To assess both dyes' response to changes in the mitochondrial membrane potential, FCCP at final concentration of 2 μM was used to artificially depolarize the mitochondrial membrane potential, and the response of the dye was monitored by time-lapse microscopy. For the duration of the experiment, the temperature was set to 37°C in the microscopy chamber. A 9-minute long time-lapse was set, so to take images every minute. Time lapse was paused just before the third frame, and 1 mL of medium solution supplemented with dye and FCCP was added. The time lapse was then restarted immediately. For the control experiments, a mock injection was made with medium and dye only.

The key experimental data associated with Figs. 1, 2, and 3 are made available on our laboratories' GitHub repository: https://github.com/OSS-Lab/ThT_CellularDistribution_ImagesAndModel.

Image analysis

Image segmentation was achieved using Fiji/ImageJ (National Institutes of Health) (44) where the cytoplasmic region for each cell was manually selected, and the mean fluorescence intensity values were obtained. Figures were created using MATLAB (The MathWorks). Fluorescence data from time-lapse experiments was obtained as above and then normalized for each cell by the average mean fluorescence of the first three frames, before the addition of FCCP. The boxplots were generated by plotting the ratio between the fluorescence of the first frame and last frame, and the individual data points were plotted using the plot spread points (beeswarm plots) algorithm (45). For colocalization analysis, both ThT and TMRM fluorescence data were normalized using a min/max normalization. A scatter plot was generated to which a linear model was fit, and the Pearson correlation coefficient was calculated using the appropriate MATLAB functions.

Mathematical model

The model presented here considered the cell as a two-compartment system separated by membranes. Thus, our abstract cell has a mitochondrial and a cytosolic compartment and is separated from the extracellular environment by a plasma membrane. The model accounted for potential-driven, passive diffusion of two cationic dyes across these compartments, using the Goldman-Hodgkin-Katz flux equation (33,34). For ThT, the model incorporated binding in both compartments and subsequent photosensitization as a combined, single reaction. Finally, the model assumed a simple, linear relation between photosensitized mitochondrial ThT level and $\Delta\Psi$m. The model thus consisted of the following ordinary differential equations describing the temporal dynamics of mitochondrial and cytosolic TMRM ($TMRM_m$ and $TMRM_c$) and mitochondrial and cytosolic free and bound, photosensitized ThT (ThT_m, ThT_c, ThT_{cphoto}, and ThT_{mphoto}):

$$\frac{dThT_c}{dt} = -Jc_{ThT} + Jm_{ThT} - Kon_c \cdot \frac{{ThT_c}^n}{{K_c}^n + {ThT_c}^n} + Koff_c \cdot ThT_{cphoto},$$

$$\frac{dThT_m}{dt} = -Jm_{ThT} - Kon_m \cdot \frac{{ThT_m}^n}{{K_m}^n + {ThT_m}^n} + Koff_m \cdot ThT_{mphoto},$$

$$\frac{dThT_{cphoto}}{dt} = Kon_c \cdot \frac{{ThT_c}^n}{{K_c}^n + {ThT_c}^n} - Koff_c \cdot ThT_{cphoto},$$

$$\frac{dThT_{mphoto}}{dt} = Kon_m \cdot \frac{{ThT_m}^n}{Km^n + {ThT_m}^n} - Koff_m \cdot ThT_{mphoto},$$

$$\frac{dTMRM_c}{dt} = -Jc_{TMRM} + Jm_{TMRM},$$

$$\frac{dTMRM_m}{dt} = -Jm_{TMRM},$$

where the passive, potential-driven fluxes of ThT and TMRM across the plasma and mitochondria membranes are described by the terms denoted by Jc_{ThT} and Jm_{ThT} and by Jc_{TMRM} and Jm_{TMRM}, respectively. The binding and photosensitization of ThT in the cytoplasm and mitochondria are both modeled using a cooperative Hill function where n is the cooperativity coefficient. K_c and K_m are the ThT concentrations producing half saturation in cytosol and mitochondria, respectively, and Kon_c and Kon_m are the maximal rate of binding and photosensitization in these two compartments. The dissociation/desensitization of cytoplasmic and mitochondrial

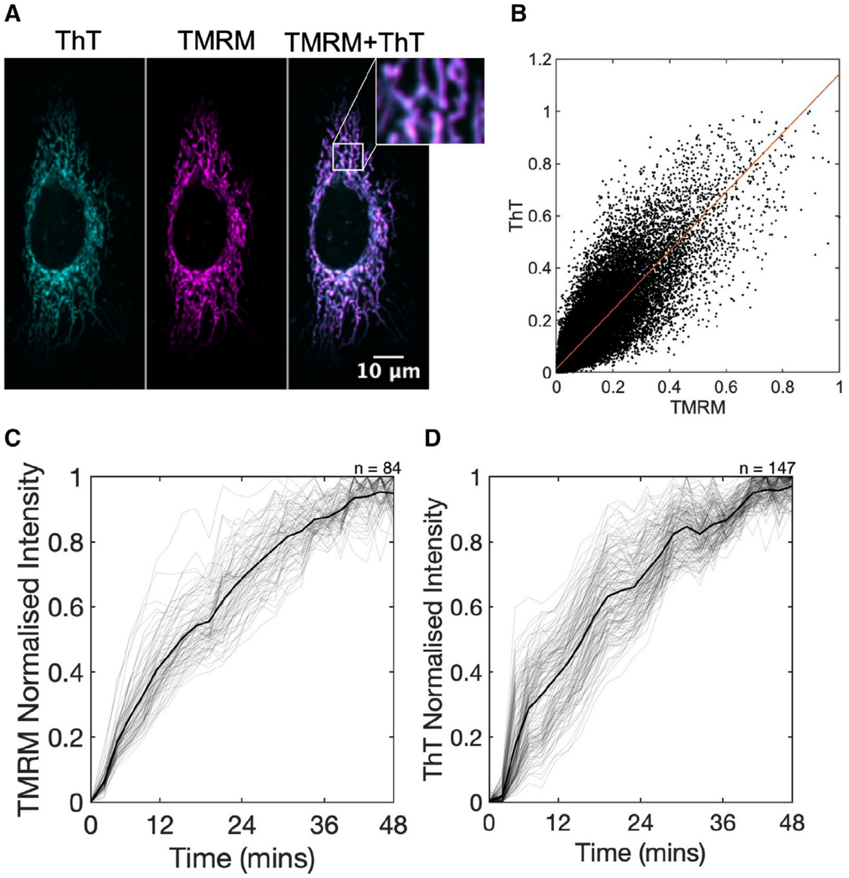

FIGURE 1 (*A*) Separate and overlayed images of a ThT- (0.2 μM) and TMRM- (25 nM) stained HeLa cell. The insert shows an enlarged section of the mitochondrial network. The color scheme, as a function of pixel intensity, is applied afterward as an aid to the eye. The scale bar applies to all images. (*B*) Correlation of pixel-wise fluorescence intensity from TMRM and ThT images and a corresponding fit using a linear model shown as a red line ($R^2 = 0.8858$). (*C* and *D*) The equilibration of TMRM (25 nM) (*C*) and ThT (0.2 μM) (*D*) fluorescence over time. Dye is added 2 mins into imaging, and fluorescence data was min-max normalized. On each panel, the gray and black lines show the fluorescence intensity of single cells and the population mean, respectively. Data are from three independent experiments, each with three technical repeats. Number of cells analyzed is shown in top-right corner.

bound ThT is determined by the dissociation constants, $Koff_c$ and $Koff_m$ respectively.

The generic form of the Goldman-Hodgkin-Katz flux equation is used to determine the Jc_{ThT} and Jm_{ThT} and by Jc_{TMRM} and Jm_{TMRM}. As an example, the flux equations is given here for ThT:

$$J = P_S Z_S^2 \frac{\Delta \Psi F^2}{RT} \frac{[ThT]_i - [ThT]_o \, exp(-Z_S F/RT)}{1 - exp(-Z_S F/RT)},$$

where the indices *i* and *o* refer to the mitochondria and cytosol (in the case of mitochondrial flux) and cytosol and the cell exterior (in the case of plasma flux). P_S and Z_S denote the permeability and charge of the modeled dye, and *F*, *R*, and *T* denote the Faraday constant, the gas constant, and the temperature.

Finally, the relation between mitochondrial bound, photosensitized ThT level and ΔΨm is modeled through a linear function:

$$\Delta\Psi\text{m} = \delta * [ThT_{mphoto}] + \Delta\Psi_{m,0},$$

where δ is a scaling parameter, and $\Delta\Psi_{m,0}$ is the basal mitochondrial membrane potential.

The parameters used to simulate the system are provided in the MATLAB files, which are made available on our laboratories' GitHub repository: https://github.com/OSS-Lab/ThT_CellularDistribution_ImagesAndModel.

RESULTS

ThT distributes in mitochondria and responds to changes in mitochondrial membrane potential

To evaluate the membrane potential dependency of ThT distribution at the single-cell level, we imaged HeLa cells with ThT by scanning confocal microscopy (see materials and methods). ThT fluorescence showed a network pattern that was reminiscent of mitochondria (Fig. 1 *A*). To check if ThT localizes in the mitochondria, we co-stained HeLa cells with the well-established mitochondrial membrane potential indicator TMRM and found that the spatial distribution of ThT overlapped with that of TMRM (Fig. 1 *A* and *B*). Both dyes also exhibited a characteristic equilibration curve with a similar equilibration profile (Fig. 1 *C* and *D*). This co-localization with TMRM and the cationic nature of ThT suggest that ThT distributes itself according to the electrical potential differences within the cell and therefore might respond to changes in the ΔΨm.

To test the membrane potential dependency of ThT localization in the mitochondria, we recorded ThT distribution under chemical perturbation of

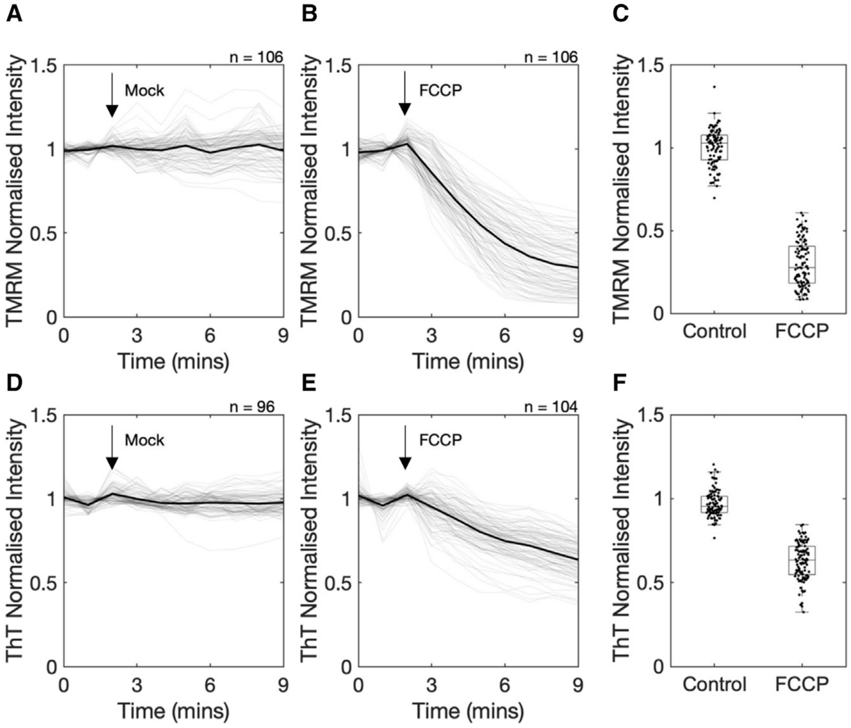

FIGURE 2 (*A, B, D,* and *E*). The temporal response of cellular TMRM (*A* and *B*) and ThT (*D* and *E*) fluorescence. Cells were incubated with either 25 nM TMRM or 0.2 μM ThT, and their fluorescence was normalized to that from the first image. At the time point indicated with an arrow, either cell culture media, as control (*A* and *D*) or media containing 2 μ M FCCP (*B* and *E*), is added. On each panel, the gray lines show the fluorescence intensity from single cells, and the black line shows the population mean. Data are collated from three independent experiments, each with three technical repeats. Number of cells analyzed is shown in top-right corner of each panel. (*C* and *F*) Boxplot of final fluorescence intensity from the cell population (time point 9 min.) shown on (*A*) and (*B*). The means of the two distributions are statistically significant (U test, $p < 0.05$) in both panels.

$\Delta\Psi$m by FCCP, a protonophore that increases the proton conductivity across the inner mitochondrial membrane and thereby collapses $\Delta\Psi$m. It has been shown that FCCP treatment results in the loss of TMRM fluorescence from the mitochondria within 5–10 min (26,27). Following the previously established experimental procedure, we performed fluorescence time-lapse microscopy of HeLa cells for 9 min, during which 2 μM FCCP was added to the medium at 2 min into the experiment. We then quantified the fluorescence intensity of TMRM over time for individual cells and plotted the rate of change from pre-FCCP levels. As seen with a previous study that was conducted on a different cell line (27), addition of FCCP caused a reduction in TMRM fluorescence, whereas mock control did not show any significant changes (Fig. 2 *A–C*). Having confirmed the dynamics with TMRM in our experimental setup and with HeLa cells, we repeated this experiment with ThT. The fluorescence intensity of ThT displayed similar dynamics as with TMRM, with fluorescence showing a decrease after the addition of FCCP (Fig. 2 *D–F*). There was less of a decrease in ThT fluorescence with FCCP addition, which could be explained by a higher degree of nonspecific binding to mitochondrial components with ThT than with TMRM. We note that nonspecific binding of cationic dyes to mitochondria has been reported before, even for TMRM, but it does not limit usability as $\Delta\Psi$m indicator since it can be accounted for in experimental design (27).

ThT at high concentrations depolarizes mitochondrial membrane potential

Previous studies have suggested that the nonspecific mitochondrial binding of membrane potential dyes, especially when used at high concentrations, could dissipate $\Delta\Psi$m (24). We also noticed that at higher concentrations, the spatial distribution of ThT changes, which could indicate that we are affecting the $\Delta\Psi$m at these concentrations (Fig. S1 *A*). To test the possible impact of ThT on $\Delta\Psi$m, we cultured cells with media containing different concentrations of ThT between 0.2 and 5 μM while monitoring $\Delta\Psi$m dynamics over time with TMRM (Fig. 3 *A*). TMRM was used at a low concentration (25 nM) to ensure that TMRM itself does not influence the $\Delta\Psi$m (24). Cells were incubated with the dyes for 1 h at 37°C, and both TMRM and ThT were imaged every 15 secs for 20 mins. At a ThT concentration of 0.2 μM, we saw no change in $\Delta\Psi$m, as monitored by TMRM fluorescence (Fig. 3 *A*, dashed line; see also Fig. S2). However, at ThT concentrations of 1 and 5 μM, a decrease in TMRM fluorescence was observed, indicating depolarization of mitochondria and loss of $\Delta\Psi$m (Fig. 3 *A*, dash-dot and dotted lines; see also Fig. S2). The time it takes for the mitochondrial membrane to

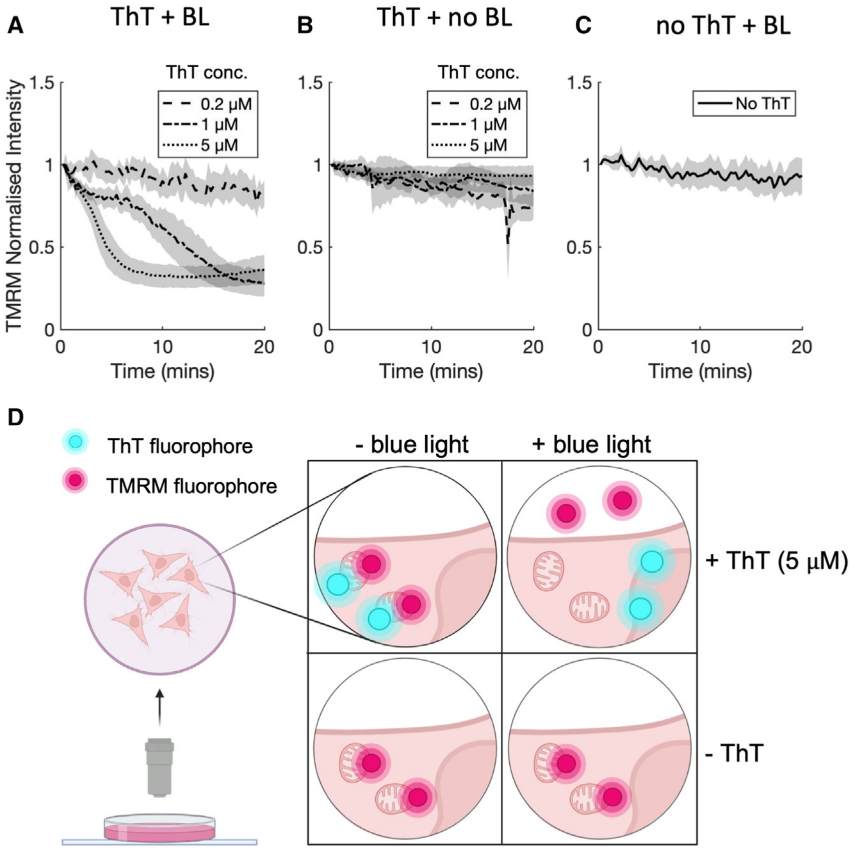

FIGURE 3 (*A* and *B*) Temporal TMRM fluorescence from cells incubated with TMRM (25 nM) and co-incubated with 0.2, 1, and 5 μM ThT and excited by both 405- and 561-nm lasers (*A*) or only the 561-nm laser (*B*). On each panel, the lines show the mean fluorescence intensity with the shaded section showing population standard deviation, SD (mean ± SD). Data are collated from three independent experiments, each with three technical repeats resulting in a total of 176 and 177 cells analyzed in (*A*) and (*B*), respectively. (*C*) Temporal TMRM fluorescence from cells incubated with TMRM (25 nM) without ThT co-staining and excited by both 405- and 561-nm lasers. Experiment design is the same as for (*A*) and (*B*), resulting in a total of 62 cells. (*D*) Cartoon depicting the setup for the co-staining experiments. Cells were incubated with either TMRM only or with TMRM and ThT and then imaged with either the 561-nm laser only or by both 405- and 561-nm lasers. Cartoon was created with BioRender.com.

depolarize was shorter at higher ThT concentrations (Fig. 3 *A*). At ThT concentrations, where a loss of ΔΨm, and TMRM leaving the mitochondria, is observed, we found that ThT also leaves the mitochondria and localizes in the nucleoli (Fig. S3 *C*). All together, these results show that, whereas ThT at concentrations of ≤0.2 μM does not affect the membrane potential and can be used to monitor ΔΨm, its application for time-lapse imaging at higher concentration may cause loss of ΔΨm.

Mitochondria depolarization by ThT results from a combination of its concentration and extent of blue light exposure

The dissipation of ΔΨm in the above experiments could be due to excitation of ThT since it has been shown that photoexcited ThT can result in the formation of oxygen radicals (28,29,30) and affect specific cellular processes (31,32). To explore this possibility, we designed additional experiments to examine the impact of the extent of light exposure on ΔΨm dissipation by ThT. Specifically, we incubated cells for 1 h with ThT and TMRM but in darkness, using different ThT concentrations. When cells were imaged at the end of this period, we found no loss in TMRM fluorescence (hence no change in ΔΨm), regardless of the ThT concentration (Fig. S4). We then repeated the co-incubation, time-lapse experiment, but we imaged TMRM only, for 20 mins (i.e., no blue light exposure). We took ThT images only at the beginning and end of this time-lapse experiment, to assess the ThT distribution. Over the course of the time lapse, TMRM fluorescence was stable (Fig. 3 *B*). These two experiments together show that the presence of ThT in the cell, even at high concentration, on its own does not cause a loss of ΔΨm. We then tested whether blue light exposure during our imaging is sufficient on its own to cause membrane depolarization. Cells were incubated with TMRM only and excited by both 405- and 561-nm lasers every 15 secs for 20 min, as above. We again found no effect on TMRM fluorescence, indicating a stable ΔΨm under blue light exposure alone (Fig. 3 *C*). We also determined that 5 μM ThT or the blue light used in our imaging condition, on their own, had no significant effect on cell viability (Fig. S5).

These results strongly suggest that when using ThT for monitoring ΔΨm, excessive imaging conditions and/or high concentrations must be avoided because a high enough concentration of photosensitized ThT causes a loss of ΔΨm.

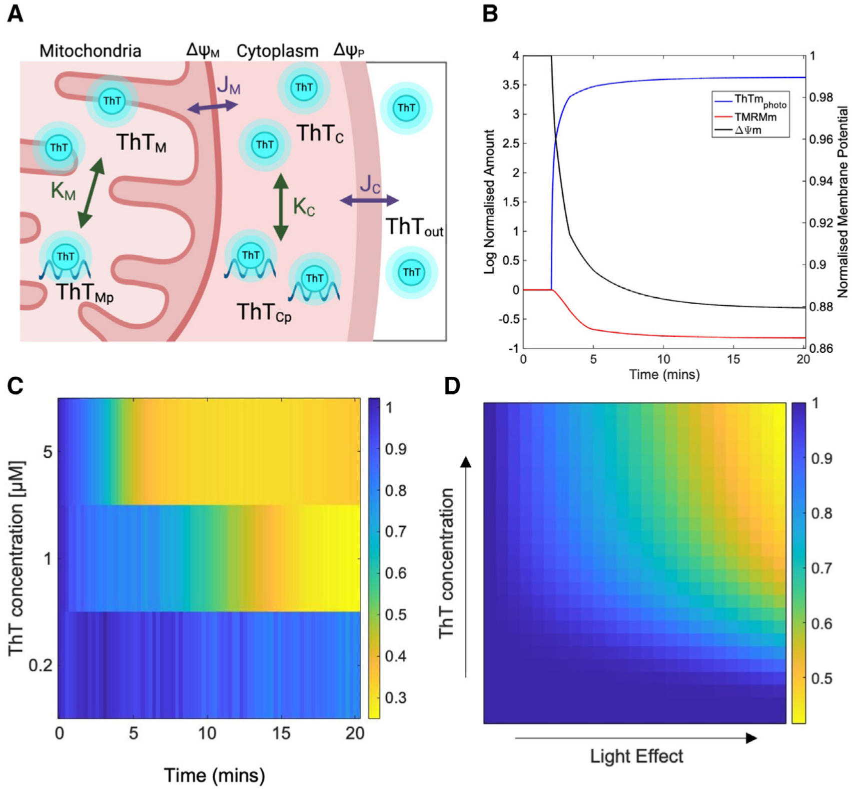

FIGURE 4 (*A*) Cartoon depicting the mathematical model, which features two compartments as mitochondria and cytosol. The different simulated variables are shown as described in the main text. Arrows indicate photosensitization events and dye flux across membranes. Cartoon was created with BioRender.com. (*B*) Normalized concentrations of simulated variables of the model over time. Simulation is run to steady state and then the system is perturbed at t = 2 min. by increasing the parameters Kon_m and Kon_c. (*C*) Phase plot showing the fold change in TMRM fluorescence (color coded) over time (x-axis), during time-lapse experiments with different levels of ThT (y-axis). These data are the same as shown in Fig. 3 *A* but are compiled here into a phase plot to allow easier comparison to simulations. (*D*) Phase plot showing normalized steady-state TMRM level (color coded) from simulations performed with different levels of ThT photosensation (x-axis, mimicking increasing light exposure) and different amounts of ThT (y-axis).

ThT and blue light effects are captured by a mechanistic mathematical model

To understand the conditions that influence membrane potential so that they can be better avoided when using ThT as a membrane potential dye, we developed a simple mechanistic model (see Material and methods and Supplementary File 1 and 2). The aim of the model was to formalize the key hypotheses needed to reproduce the experimental observation of the combined need for blue light and ThT to cause depolarization of the mitochondrial membrane potential. To this end, several pieces of evidence suggest that when bound ThT is excited, singlet oxygen is produced (28,29,30). This includes our own investigations where ascorbic acid, a ROS scavenger, was able to slow the loss of ΔΨm, under ThT and blue light condition (Fig. S7). We therefore assumed that upon light irradiation, ThT that is nonspecifically bound becomes excited and leads to ROS production. This ROS production, in turn, can cause a depolarization of mitochondria and loss of ΔΨm, thereby resulting in TMRM and ThT leaving the mitochondria as observed in our experiments.

To incorporate these assumptions in the model, we considered a combined reaction capturing nonspecific binding and photosensitization of ThT in the mitochondria (ThT_{mphoto}) and cytosol (ThT_{cphoto}), with the rate of reaction given by a Hill function. We also implemented a linear relation between ThT_{mphoto} concentration and disruption of ΔΨm (see materials and methods). In addition, we implemented passive flux of ThT and TMRM across both plasma and mitochondrial membranes, using the Goldman-Hodgkin-Katz flux equation (33,34) (Fig. 4 *A*). Using this model, we simulated the dynamics of both ThT and TMRM distribution in the cell. When we simulate conditions that mimic absence of blue light (low maximal rate of ThT_{mphoto} formation), the dye equilibration does not impact ΔΨm significantly, which reaches a steady state close to its initial value set at the beginning of the simulation (Fig. 4 *B*, $t < 2$ min.). When we simulate high blue light (high maximal rate of ThT_{mphoto} formation), there is an increase in the amount of both ThT_{mphoto} and ThT_{cphoto} (Fig. 4 *B*, simulation traces after $t = 2$ min.). These dynamic changes in ThT distribution cause a depolarization of ΔΨm, reaching a new steady state at a less negative potential, and consequently of the TMRM leaving the cell (Fig. 4 *B*).

Our aim was to then observe if this model can recapture our experimental results as seen in Fig. 3 *A*. To allow for the comparison for experimental and simulation results, the experimental data in Fig. 3 *A* are shown as a phase plot in Fig. 4 *C*, where the effect on TMRM fluorescence (color bar) at different concentrations of

ThT (y-axis) and different degrees of blue light exposure, which is analogous to time (x-axis), is shown. We then ran simulations with different concentrations of ThT and at different maximal photosensitization rates, mimicking different light exposure levels to create a similar phase plot for comparison with our experimental data (Fig. 4 *C*). The resulting phase plot for the system dynamics (Fig. 4 *C*) shows that $\Delta\Psi$m is dependent on both ThT level and light exposure, as seen experimentally when using TMRM fluorescence as an indicator of $\Delta\Psi$m (Fig. 4 *D*). The key parameters controlling the phase plot obtained from the model (shown in Fig. 4 *C*) are those relating to the Hill function, which dictate the dynamics of ThT binding and photosensitization. To test the robustness of the model, we performed a sensitivity analysis to determine the effect that these major parameters have on the phase plot. We determined that both the coefficient of nonlinearity (*N*) and the saturation concentrations (K_m and K_c,) affect the threshold of ThT concentration and blue light required for any changes to $\Delta\Psi$m (see Figs. S8 and S9).

DISCUSSION

We investigated the possibility that ThT, a commonly used protein-aggregate marker, also acts as $\Delta\Psi$m indicator in a mammalian cell. Using live single-cell microscopy, we show that ThT can distribute itself in mitochondria in a $\Delta\Psi$m-dependent manner and that it responds to mitochondrial membrane depolarization by leaving mitochondria. Both responses show similar qualitative dynamics to a well-established $\Delta\Psi$m indicator, TMRM. Additionally, we found that cell equilibration dynamics of TMRM and ThT are similar.

We also investigated the potential of ThT to dissipate $\Delta\Psi$m at higher concentrations, similar to other cationic dyes. We found that a high concentration of ThT alone was not responsible for the loss of $\Delta\Psi$m, but rather this effect was due to both the presence of ThT and its photosensitization by blue light. Previous studies have demonstrated that when bound ThT becomes photosensitized, this can lead to ROS production, which could lead to the loss of $\Delta\Psi$m. Indeed, we find that ROS scavengers can slow the impact of combined ThT and blue light. Further, a simple mechanistic model incorporating ThT photosensitization and impact on $\Delta\Psi$m could qualitatively recapitulate the experimental observations. With this model, we demonstrate that if high concentrations and harsher imaging conditions are avoided, then ThT can be used as a membrane potential indicator in mammalian cells.

ThT has been used for live-cell time-lapse imaging of protein aggregates (e.g., (35,36,37)). Our finding, that the combination of ThT presence and the blue light exposure for imaging ThT fluorescence could impact $\Delta\Psi$m, raises a cautionary note against the use of ThT in live-cell imaging for assessment of protein aggregates or DNA. Particularly, we note that changes in $\Delta\Psi$m could alter cellular ATP levels, which is shown to interlink to the prevention of protein aggregation through ATP's destabilizing effects on aggregates (38,39). This suggests that changes in $\Delta\Psi$m, caused by ThT presence and blue light imaging, can then feed back to alter protein aggregation levels. Therefore, the interpretations of live-cell ThT microscopy, regardless of whether it is aimed to study protein aggregates or $\Delta\Psi$m, would need careful consideration of the possible effects arising from mitochondrial membrane potential depolarization. We note that neither ThT nor blue light on their own was found to impact $\Delta\Psi$m in our experiments, nor cell viability, suggesting that ThT can still be utilized for live-cell studies when it is conducted with low concentration of ThT and with low-exposure imaging modalities.

In summary, we have shown that ThT can act as a $\Delta\Psi$m indicator and that ThT and blue light exposure together can be used to collapse $\Delta\Psi$m. The former finding leads to the question as to whether ThT should be used over other available $\Delta\Psi$m indicators, such as TMRM. We find both dyes show similar equilibration profiles, but ThT seems to have a higher unspecific binding potential and requires light at lower wavelengths to be excited. These points make it possibly less attractive as a $\Delta\Psi$m indicator compared with TMRM. However, we also note that microfluidic channels made from polydimethylsiloxane are shown to bind TMRM and thereby cause a high background signal, making it hard to distinguish between cells and background (40). ThT has been previously used in microfluidics and does not present the same issues (13,41,42). Thus, microfluidic studies could be one area where ThT can be utilized as an alternative to TMRM, opening a potential opportunity to take $\Delta\Psi$m measurements of single mammalian cells in microfluidic devices. The second finding listed above seems to be a unique feature to ThT and possibly relates to its potential to generate ROS. This feature opens an interesting avenue of study in that ThT and pixel-level controlled blue light can be utilized together to study membrane depolarization dynamics at the single-mitochondrial level.

SUPPORTING MATERIAL

Supporting material can be found online at https://doi.org/10.1016/j.bpr.2023.100134.

AUTHOR CONTRIBUTIONS

O.S.S., M.A., and E.S. have devised the study. E.S. and Z.S. performed experiments. E.S., H.D., and O.S.S. performed analyses and simulations. E.S., M.A., and O.S.S. analyzed and interpreted the results and wrote the manuscript.

ACKNOWLEDGMENTS

We would like to thank Dr. Ian Hands-Portman for his microscopy training and technical assistance.

This project is funded by the Biotechnology and Biological Sciences Research Council (BBSRC) (grant IDs BB/T010150/1 and BB/M017982/1) and University of Warwick funded Midlands Integrative Biosciences Training Partnership (MIBTP) (grant ID BB/M01116X/1) and a Cancer Research UK (CRUK) Pioneer Award (to O.S.S.).

DECLARATION OF INTERESTS

The authors declare no competing interests.

REFERENCES

1. Gade Malmos, K., L. M. Blancas-Mejia, ..., D. Otzen. 2017. ThT 101: a primer on the use of thioflavin T to investigate amyloid formation. *Amyloid.* 24:1–16. https://doi.org/10.1080/13506129.2017.1304905.
2. Xing, Y., and K. Higuchi. 2002. Amyloid bril proteins. *Mech. Ageing Dev.* 123:1625–1636. https://doi.org/10.1016/s0047-6374(02)00098-2.
3. Xue, C., T. Y. Lin, ..., Z. Guo. 2017. Thioflavin T as an amyloid dye: Fibril quantification, optimal concentration and effect on aggregation. *R. Soc. Open Sci.* 4:160696. https://doi.org/10.1098/rsos.160696.
4. Groenning, M., M. Norrman, ..., S. Frokjaer. 2007. Binding mode of Thioflavin T in insulin amyloid fibrils. *J. Struct. Biol.* 159:483–497. https://doi.org/10.1016/j.jsb.2007.06.004.
5. Lockhart, A., L. Ye, ..., J. Brown. 2005. Evidence for the presence of three distinct binding sites for the thioflavin T class of Alzheimer's disease PET imaging agents on β-amyloid peptide fibrils. *J. Biol. Chem.* 280:7677–7684. https://doi.org/10.1074/jbc.M412056200.
6. Maezawa, I., H. S. Hong, ..., L. W. Jin. 2008. Congo red and thioflavin-T analogs detect Aβ oligomers. *J. Neurochem.* 104:457–468. https://doi.org/10.1111/j.1471-4159.2007.04972.x.
7. Voropai, E. S., M. P. Samtsov, ..., V. N. Uverskii. 2003. Spectral properties of thioflavin T and its complexes with amyloid fibrils. *J. Appl. Spectrosc.* 70:868–874. https://doi.org/10.1023/B:JAPS.0000016303.37573.7e.
8. Stsiapura, V. I., A. A. Maskevich, ..., I. M. Kuznetsova. 2007. Computational study of thioflavin T torsional relaxation in the excited state. *J. Phys. Chem. A.* 111:4829–4835. https://doi.org/10.1021/jp070590o.
9. Biancalana, M., K. Makabe, ..., S. Koide. 2009. Molecular Mechanism of Thioflavin-T Binding to the Surface of β-Rich Peptide Self-Assemblies. *J. Mol. Biol.* 385:1052–1063. https://doi.org/10.1016/j.jmb.2008.11.006.
10. Biancardi, A., T. Biver, ..., M. Venturini. 2014. Mechanistic aspects of thioflavin-T self-aggregation and DNA binding: Evidence for dimer attack on DNA grooves. *Phys. Chem. Chem. Phys.* 16:20061–20072. https://doi.org/10.1039/c4cp02838d.
11. Xu, S., Q. Li, ..., Y. Tang. 2016. Thioflavin T as an efficient fluorescence sensor for selective recognition of RNA G-quadruplexes. *Sci. Rep.* 6:24793. https://doi.org/10.1038/srep24793.
12. Khurana, R., C. Coleman, ..., S. Singh. 2005. Mechanism of thioflavin T binding to amyloid fibrils. *J. Struct. Biol.* 151:229–238. https://doi.org/10.1016/j.jsb.2005.06.006.
13. Prindle, A., J. Liu, ..., G. M. Süel. 2015. Ion channels enable electrical communication in bacterial communities. *Nature.* 527:59–63. https://doi.org/10.1038/nature15709.
14. Stratford, J. P., C. L. A. Edwards, ..., M. Asally. 2019. Electrically induced bacterial membrane-potential dynamics correspond to cellular proliferation capacity. *Proc. Natl. Acad. Sci. USA.* 116:9552–9557. https://doi.org/10.1073/pnas.1901788116.
15. Mancini, L., G. Terradot, ..., T. Pilizota. 2020. A General Workflow for Characterization of Nernstian Dyes and Their Effects on Bacterial Physiology. *Biophys. J.* 118:4–14. https://doi.org/10.1016/j.bpj.2019.10.030.
16. Sirec, T., J. M. Benarroch, ..., M. Asally. 2019. Electrical Polarization Enables Integrative Quality Control during Bacterial Differentiation into Spores. *iScience.* 16:378–389. https://doi.org/10.1016/j.isci.2019.05.044.
17. Geissler, A., T. Krimmer, ..., N. Pfanner. 2000. Membrane potential-driven protein import into mitochondria: The sorting sequence of cytochrome b2 modulates the Δψ-dependence of translocation of the matrix-targeting sequence. *Mol. Biol. Cell.* 11:3977–3991. https://doi.org/10.1091/mbc.11.11.3977.
18. Lo, C. J., M. C. Leake, ..., R. M. Berry. 2007. Nonequivalence of membrane voltage and ion-gradient as driving forces for the bacterial flagellar motor at low load. *Biophys. J.* 93:294–302. https://doi.org/10.1529/biophysj.106.095265.
19. Sundaram, G. S. M., K. Binz, ..., V. Sharma. 2018. Live-cell fluorescence imaging: Assessment of thioflavin T uptake in human epidermal carcinoma cells. *MedChemComm.* 9:946–950. https://doi.org/10.1039/c8md00101d.
20. Yang, F., D. Yang, ..., Y. Tang. 2023. An inner salt derivative of thioflavin t designed for live-cell imaging of mitochondrial G-Quadruplexes. *Sensor. Actuator. B Chem.* 374, 132820. https://doi.org/10.1016/j.snb.2022.132820.
21. Zhang, S., H. Sun, ..., Y. Tang. 2018. Direct visualization of nucleolar G-quadruplexes in live cells by using a fluorescent light-up probe. *Biochim. Biophys. Acta Gen. Subj.* 1862:1101–1106. https://doi.org/10.1016/j.bbagen.2018.01.022.
22. Kamo, N., M. Muratsugu, ..., Y. Kobatake. 1979. Membrane potential of mitochondria measured with an electrode sensitive to tetraphenyl phosphonium and relationship between proton electrochemical potential and phosphorylation potential in steady state. *J. Membr. Biol.* 49:105–121. https://doi.org/10.1007/BF01868720.
23. Ehrenberg, B., V. Montana, ..., L. M. Loew. 1988. Membrane potential can be determined in individual cells from the nernstian distribution of cationic dyes. *Biophys. J.* 53:785–794. https://doi.org/10.1016/S0006-3495(88)83158-8.
24. Scaduto, R. C., and L. W. Grotyohann. 1999. Measurement of mitochondrial membrane potential using fluorescent rhodamine derivatives. *Biophys. J.* 76:469–477. https://doi.org/10.1016/S0006-3495(99)77214-0.
25. Rottenberg, H. 1984. Membrane potential and surface potential in mitochondria. Uptake and Binding of Lipophilic Cations. *J. Membr. Biol.* 81:127–138. https://doi.org/10.1016/0005-2728(84)90140-3.
26. Farkas, D. L., M. D. Wei, ..., L. M. Loew. 1989. Simultaneous imaging of cell and mitochondria membrane potentials. *Biophys. J.* 56:1053–1069.
27. Nicholls, D. G. 2012. Fluorescence measurement of mitochondrial membrane potential changes in culture cells. *Mitochondrial Bioenergetics: Methods and Protocols (Methods in Molecular*

Biology. 810:103–117. https://doi.org/10.1007/978-1-61779-382-0.

28. Bondia, P., J. Torra, …, C. Flors. 2020. Nanoscale View of Amyloid Photodynamic Damage. *J. Am. Chem. Soc.* 142:922–930. https://doi.org/10.1021/jacs.9b10632.
29. Ni, J., Y. Wang, …, B. Z. Tang. 2021, January 2. Aggregation-induced generation of reactive oxygen species: Mechanism and photosensitizer construction. *Molecules.* 26, 268. https://doi.org/10.3390/molecules26020268.
30. Taniguchi, A., Y. Shimizu, …, M. Kanai. 2016. Switchable photooxygenation catalysts that sense higher-order amyloid structures. *Nat. Chem.* 8:974–982. https://doi.org/10.1038/nchem.2550.
31. Ahn, M., B. I. Lee, …, C. B. Park. 2019. Chemical and mechanistic analysis of photodynamic inhibition of Alzheimer's beta-amyloid aggregation. *Chem. Commun.* 55:1152–1155. https://doi.org/10.1039/C8CC09288E.
32. Bondia, P., C. Flors, and J. Torra. 2021. Boosting the inactivation of bacterial biofilms by photodynamic targeting of matrix structures with Thioflavin T. *Chem. Commun.* 57:8648–8651. https://doi.org/10.1039/d1cc03155d.
33. Goldman, D. E. 1943. Potential, impedance, and rectification in membranes. *J. Gen. Physiol.* 27:37–60. https://doi.org/10.1085/jgp.27.1.37.
34. Hodgkin, A. L., and B. Katz. 1949. The effect of sodium ions on the electrical activity of the giant axon of the squid. *J. Physiol.* 108:37–77. https://doi.org/10.1113/jphysiol.1949.sp004310.
35. Beriault, D. R., and G. H. Werstuck. 2013. Detection and quantification of endoplasmic reticulum stress in living cells using the fluorescent compound, Thioflavin T. *Biochim. Biophys. Acta.* 1833:2293–2301. https://doi.org/10.1016/j.bbamcr.2013.05.020.
36. Caron, N. S., C. L. Hung, …, R. Truant. 2014. Live cell imaging and biophotonic methods reveal two types of mutant huntingtin inclusions. *Hum. Mol. Genet.* 23:2324–2338. https://doi.org/10.1093/hmg/ddt625.
37. Sugimoto, S., K. I. Arita-Morioka, …, T. Ogura. 2015. Thioflavin T as a fluorescence probe for monitoring RNA metabolism at molecular and cellular levels. *Nucleic Acids Res.* 43:e92. https://doi.org/10.1093/nar/gkv338.
38. Patel, A., L. Malinovska, …, A. A. Hyman. 2017. Biochemistry: ATP as a biological hydrotrope. *Science.* 356:753–756. https://doi.org/10.1126/science.aaf6846.
39. Pu, Y., Y. Li, …, F. Bai. 2019. ATP-Dependent Dynamic Protein Aggregation Regulates Bacterial Dormancy Depth Critical for Antibiotic Tolerance. *Mol. Cell.* 73:143–156.e4. https://doi.org/10.1016/j.molcel.2018.10.022.
40. Zand, K., T. Pham, …, P. J. Burke. 2013. Nanofluidic Platform for Single Mitochondria Analysis Using Fluorescence Microscopy. *Anal. Chem.* 85:6018–6025. https://doi.org/10.1021/ac4010088.
41. Saar, K. L., E. V. Yates, …, T. P. J. Knowles. 2016. Automated Ex Situ Assays of Amyloid Formation on a Microfluidic Platform. *Biophys. J.* 110:555–560. https://doi.org/10.1016/j.bpj.2015.11.3523.
42. Shammas, S. L., G. A. Garcia, …, D. Klenerman. 2015. A mechanistic model of tau amyloid aggregation based on direct observation of oligomers. *Nat. Commun.* 6:7025. https://doi.org/10.1038/ncomms8025.
43. Geraghty, R. J., A. Capes-Davis, …, M. Vias; Cancer Research UK. 2014. Guidelines for the use of cell lines in biomedical research. *Br. J. Cancer.* 111:1021–1046. https://doi.org/10.1038/bjc.2014.166.
44. Schindelin, J., I. Arganda-Carreras, …, A. Cardona. 2012. Fiji: An open-source platform for biological-image analysis. *Nat. Methods.* 9:676–682. https://doi.org/10.1038/nmeth.2019.
45. Jonas 2021. plot spread points (beeswarmplot). MATLAB Central File Exchange. https://www.mathworks.com/matlabcentral/fileexchange/37105-plot-spread-points-beeswarm-plot.

Biophysical Reports, Volume *3*

Supplemental information

Thioflavin T indicates mitochondrial membrane potential in mammalian cells

Emily Skates, Hadrien Delattre, Zoe Schofield, Munehiro Asally, and Orkun S. Soyer

SUPPORTING MATERIAL

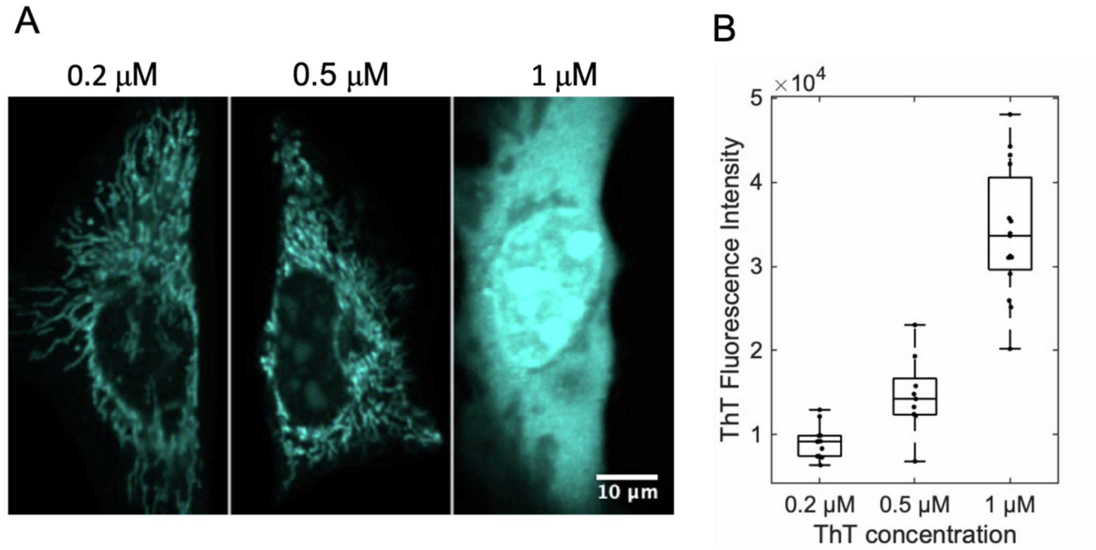

Figure S1: (A) Distribution of ThT in HeLa cells at ThT concentrations of 0.2, 0.5, and 1 μM (left to right). The scale bar applies to all images. Excitation was at 405 nm (see *Methods* for further details of imaging conditions). Each cell is representative of the total population. **(B)** Fluorescence intensity of a population of cells at ThT concentrations of 0.2, 0.5 and 1 μM. A total of 12, 9 and 15 cells were analysed at the respective concentrations. Differences among populations were statistically significant (U test, $p < 0.05$).

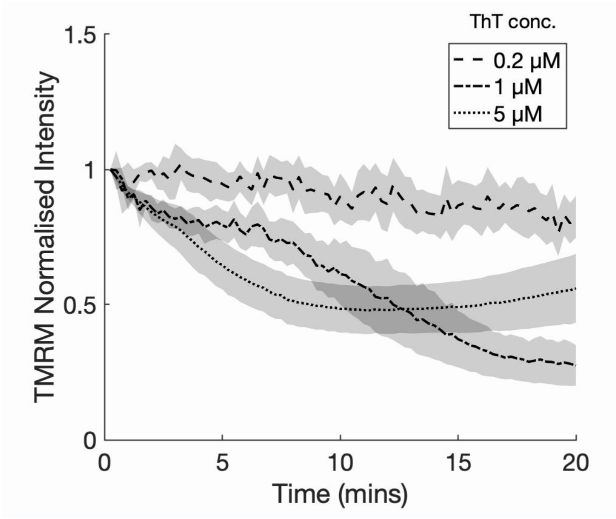

Figure S2: Shows the raw data from Fig 3B without background subtraction.

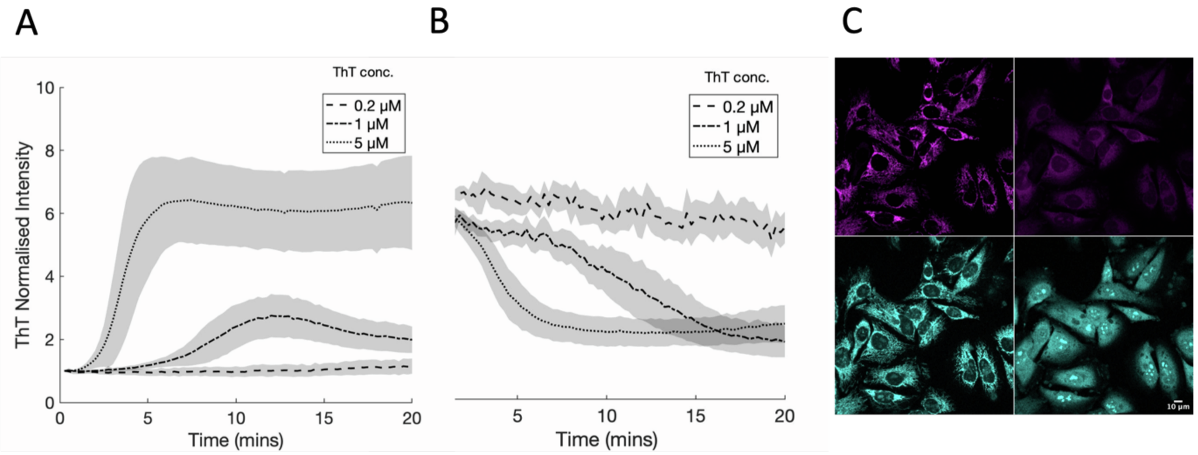

S3: Nuclear ThT fluorescence **(A)** and cellular TMRM fluorescence **(B)** for cells co-stained with 25 nM TMRM and 0.2, 1 and 5 µM ThT and imaged in red and blue light**.** On each panel, the bold lines show the mean fluorescence intensity, and the shaded section shows population standard deviation, SD (mean±SD). Data is collated from three independent experiments, each with three technical repeats resulting in a total of 177 cells analysed. **(C)** Snap shot images at the beginning and end of the experiment shown on panels A and B, with 5 µM ThT. The top two images is the TMRM fluorescence taken at the beginning (left) and end (right) of the timelapse. The two bottom images is the ThT fluorescence taken at the beginning (left) and end (right) of the timelapse.

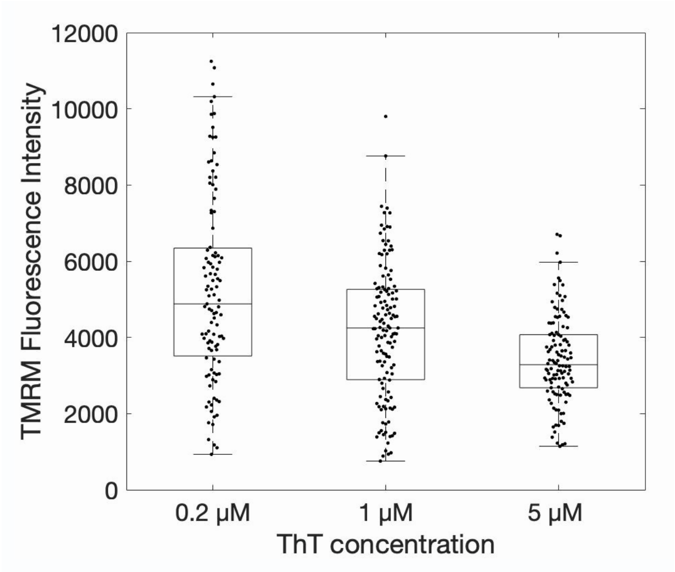

Figure S4: The response of cellular TMRM fluorescence (25 nM) after co-staining with 0.2, 1 and 5 µM ThT for 1 hr. Each data point represents one cell, with the analysed population for the three ThT concentrations totalling 107, 133, and 118 cells respectively.

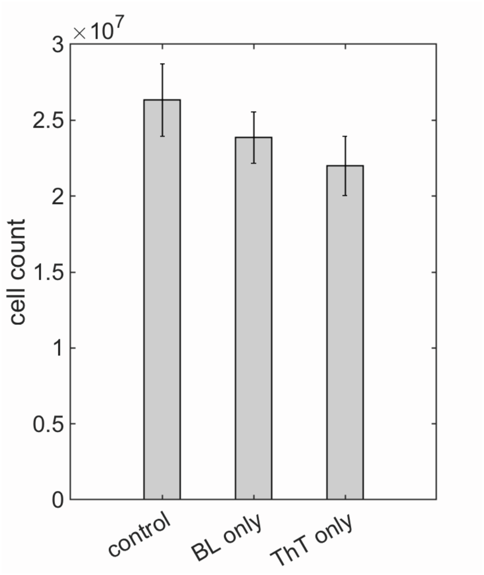

Figure S5: Cell count data 24hrs after HeLa cells were incubated on their own (control), with 5 µM ThT in the dark (ThT only), or exposed to 5 minutes of blue light (~84.7uW, BL only). There were no significant differences observed among these three experimental groups ($p < 0.05$). Data was collated from three independent experiments, each with three technical repeats.

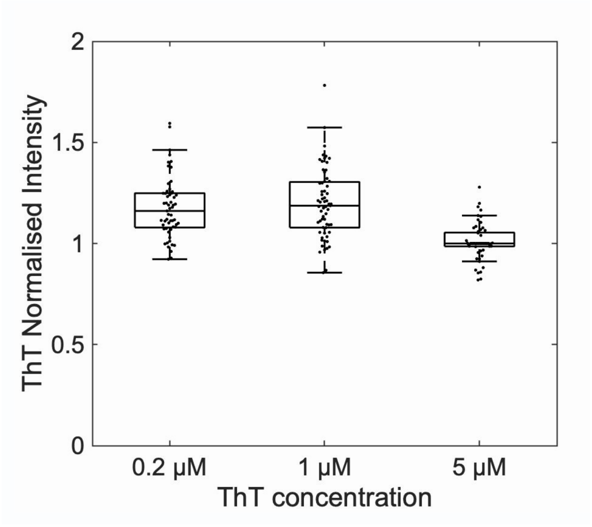

Figure S6: Cellular ThT fluorescence when cells were co-stained with TMRM (25 nM) and ThT (0.2, 1 and 5 µM). ThT fluorescence intensity ratio between before and after cells were imaged in red light for the time-lapse experiment shown in Fig 3C, i.e. beginning and end points of the experiment.

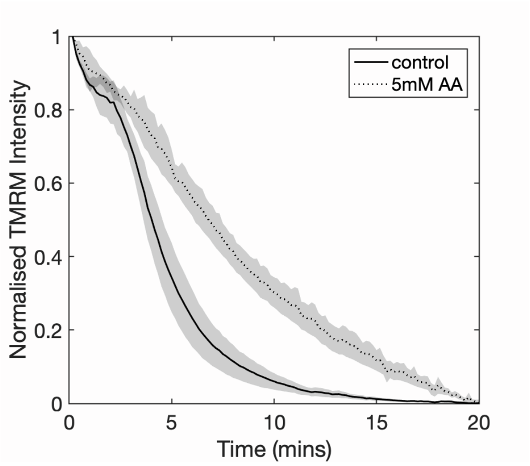

Figure S7: The effect of ascorbic acid on ThT-blue light induced TMRM signal loss. The average fluorescent trace of HeLa cells incubated with 5 µM ThT, 25 nM TMRM, and with and without 5 mM of ascorbic acid (AA). The black lines represent the mean fluorescence intensity with the shaded section showing population standard deviation, SD (mean ± SD). Data is collected from three independent experiments, each with three technical repeats resulting in a total of 137 cells for the control and 160 cells in the case of ascorbic acid treatment.

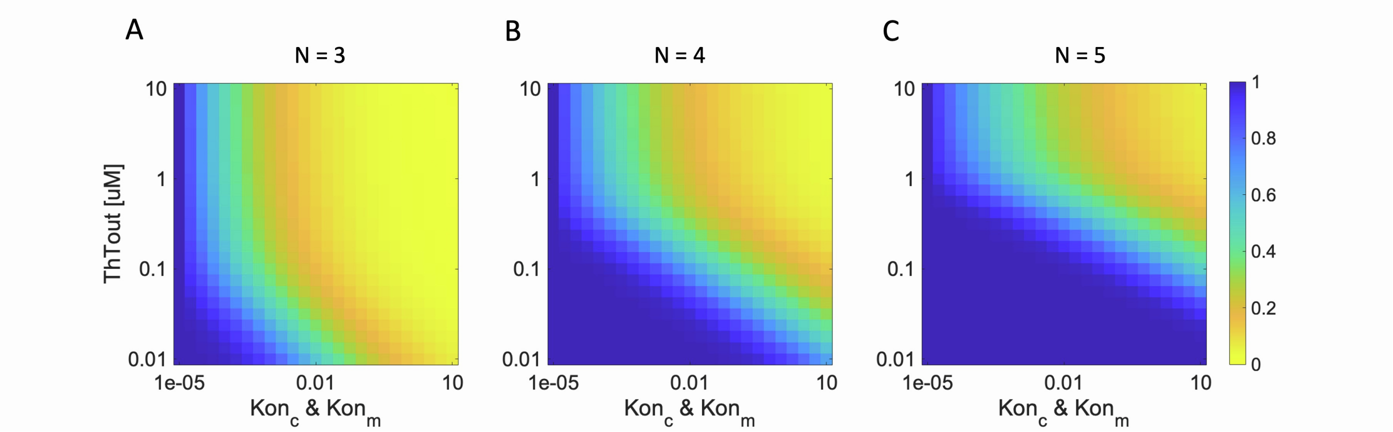

Figure S8: Phase plots showing normalised steady-state TMRM level (colour coded) from simulations performed with different levels of ThT photosensitisation (x-axis, parameters Kon_c and Kon_m, mimicking increasing photosensitisation in the cytosol and mitochondria respectively) and different amounts of ThT (y-axis, ThTout). **A-C)** Phase plot results for different values of the model parameters controlling the coefficient of nonlinearity in the Hill-function used for modelling the photosensitisation effect (*N*).

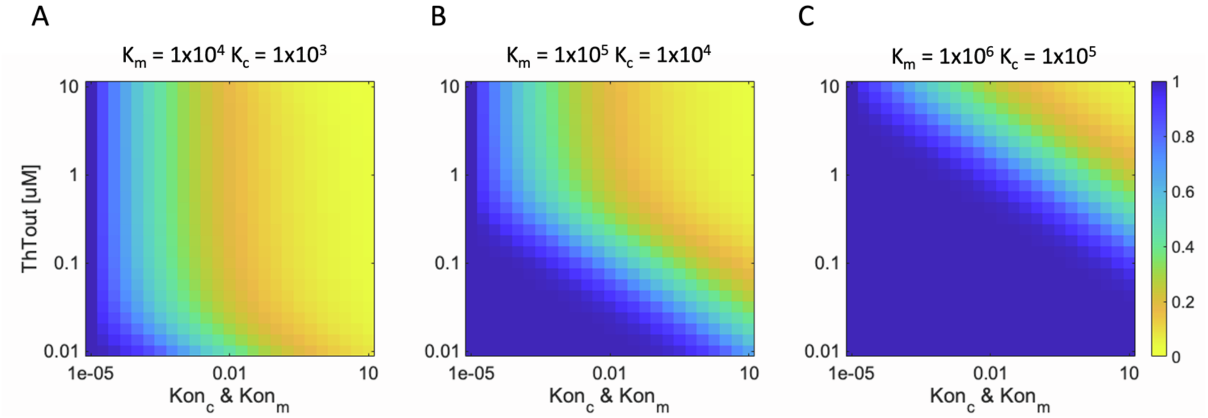

Figure S9: Phase plots showing normalised steady-state TMRM level (colour coded) from simulations performed with different levels of ThT photosensitisation (x-axis, parameters Kon_c and Kon_m, mimicking increasing photosensitisation in the cytosol and mitochondria respectively) and different amounts of ThT (y-axis, ThT_{out}). **A-C)** Phase plot results for different values of the model parameters controlling the saturation concentration for the Hill-function used for modelling the photosensitisation effect (K_m and K_c, for photosensitisation dynamics in mitochondria and cytosol respectively).
